# Early clinical experience with ⁶⁸Ga-Trivehexin PET/CT in parathyroid adenomas: a proof-of- concept imaging study

**DOI:** 10.1007/s12149-026-02232-x

**Published:** 2026-05-22

**Authors:** Mehmet Erdogan, Hakan Korkmaz, Mustafa Avcı, Zeynep Özdemir, Ahmet Tunç, Sevim Süreyya Sengul

**Affiliations:** 1 Faculty of Medicine, Department of Nuclear Medicine, Suleyman Demirel University, Isparta, Turkey; 2 Faculty of Medicine, Department of Endocrinology, Suleyman Demirel University, Isparta, Turkey

**Keywords:** ⁶⁸Ga-Trivehexin PET/CT, Parathyroid adenomas, ⁹⁹ᵐTc-Sestamibi scintigraphy

## Abstract

**Objective:**

The success of surgical treatment in Primary Hyperparathyroidism (PHPT) depends on the accurate localization of the parathyroid adenoma. Although cervical ultrasonography (USI) and ⁹⁹ᵐTc- Sestamibi scintigraphy are common methods in this context, their diagnostic performance might be limited in small, ectopically located, or biologically atypical lesions. As a novel PET agent targeting integrin αvβ6 (alpha-v beta-6), ⁶⁸Ga-Trivehexin can image biological processes associated with pathological tissue remodeling. This proof-of-concept study aimed to evaluate the feasibility and potential diagnostic contribution of ⁶⁸Ga-Trivehexin PET/CT in detecting parathyroid adenoma in patients with PHPT.

**Methods:**

This single-center, prospective proof-of-concept study included 28 patients who had a biochemical diagnosis of PHPT. All patients underwent neck ultrasound, ⁹⁹ᵐTc-Sestamibi parathyroid scintigraphy, and ⁶⁸Ga-Trivehexin PET/CT imaging. Fine Needle Aspiration Biopsy (FNAB) and Parathyroid Hormone (PTH) washout tests were performed for some patients as clinically necessary. Histopathological findings were used as the reference standard in patients who underwent surgery. The agreement between imaging methods was evaluated with Cohen’s Kappa coefficient for exploratory purposes.

**Results:**

A total of 64.2% of the patients were female, and the mean age was 56.1 ± 11.3 years. ⁹⁹ᵐTc- Sestamibi scintigraphy was found to be positive in 60.7% of the patients, while the positivity rate for ⁶⁸Ga-Trivehexin PET/CT was 78.6%. A significant proportion of cases with negative ⁹⁹ᵐTc- Sestamibi scintigraphy showed positive lesions with ⁶⁸Ga-Trivehexin PET/CT. Statistically significant, moderate agreement levels were detected in all patients between ⁹⁹ᵐTc-Sestamibi scintigraphy and ⁶⁸Ga-Trivehexin PET/CT (kappa = 0.430; p = 0.013). A moderate level of agreement was observed between the two methods in patients with histopathologically confirmed parathyroid adenoma (kappa = 0.429; p = 0.070).

**Conclusion:**

The results of this proof-of-concept study imply that ⁶⁸Ga-Trivehexin PET/CT may be a feasible and promising imaging method for localizing parathyroid adenomas in patients with primary hyperparathyroidism. It may offer potential contributions as a complementary diagnostic tool,

particularly in selected cases where conventional imaging methods are limited. Further large, multicenter studies are needed to confirm these findings.

## Introduction

As a common endocrine disease characterized by hypercalcemia and elevated parathyroid hormone (PTH), Primary Hyperparathyroidism (PHPT) is most often caused by parathyroid adenomas [1]. The curative treatment of the disease is surgery. The success of surgery depends on the accurate and reliable localization of the parathyroid adenoma, highlighting the critical importance of preoperative imaging [2]. In current clinical practice, neck ultrasound and ⁹⁹ᵐTc- Sestamibi scintigraphy (SPECT/SPECT-CT) are used as standard localization methods. However, the decrease in diagnostic sensitivity of these methods in small-sized, intrathyroidal, or ectopic parathyroid adenomas limits their effectiveness [3]. Also, parathyroid adenomas with low mitochondrial content can lead to false-negative results on scintigraphy or diagnostic difficulties because of early washout [4]. Recently, 18F-fluorocholine PET/CT has been reported to have high diagnostic performance, especially in cases that cannot be localized with conventional imaging methods [5]. On the other hand, ⁶⁸Ga-Trivehexin, currently under preclinical investigation, has emerged as a potential alternative PET radiotracer for parathyroid adenoma imaging.

⁶⁸Ga-Trivehexin is a PET agent that has been identified in recent years to target integrin receptors, particularly the αvβ6 integrin subtype, and plays a role in cell-extracellular matrix interactions. Although αvβ6 integrin shows limited expression under physiological conditions, it has been reported to increase in epithelial cell activation, tissue remodeling, and pathological proliferation processes [6]. Studies are being conducted with preliminary data on the imaging of tumors expressing the integrin αvβ6, such as head and neck squamous cell carcinoma and pancreatic ductal adenocarcinoma, using ⁶⁸Ga-Trivehexin [7]. In these studies, incidental radiotracer uptake with ⁶⁸Ga-Trivehexin has been observed in some non-oncological conditions (idiopathic pulmonary fibrosis and primary hyperparathyroidism) [8], which suggests that ⁶⁸Ga- Trivehexin may be a potential imaging tool in parathyroid adenomas. In this context, the current literature contains only limited preliminary evidence regarding the imaging of parathyroid adenomas with ⁶⁸Ga-Trivehexin.

The aim of the present study was not to claim diagnostic accuracy, but to evaluate the feasibility and diagnostic contribution of ⁶⁸Ga-Trivehexin PET/CT in detecting parathyroid adenoma in patients with primary hyperparathyroidism and to present preliminary clinical data in a proof-of-concept manner by comparing it with conventional imaging methods.

### Patients and methods

The study had a single-center, prospective, proof-of-concept design and was conducted at the Department of Nuclear Medicine, Faculty of Medicine, Süleyman Demirel University. Twenty-eight patients diagnosed with primary hyperparathyroidism because of elevated serum calcium (Ca) and parathyroid hormone (PTH) levels and referred to our department for parathyroid imaging were included in the study. All patients underwent conventional imaging methods, including neck ultrasonography (USI) and ⁹⁹ᵐTc-Sestamibi parathyroid scintigraphy. Then, ⁶⁸Ga-Trivehexin PET/ CT imaging was performed on the patients. In line with clinical necessity, some patients underwent USI-guided Fine-Needle Aspiration Biopsy (FNAB) and PTH washout test. Thirteen of the 28 patients included in the study underwent surgical excision based on clinical necessity and multidisciplinary evaluation. Histopathological examination results of the excised tissues were accepted as the reference standard in the patients who underwent surgery. Since the patients did not undergo surgery, FNAB cytology results and/or PTH washout results were used for additional diagnostic confirmation. Patients diagnosed with parathyroid adenoma, showing parathyroid cells in FNAB cytology results, and having high parathyroid hormone levels following the washout were separately compared with ⁹⁹ᵐTc-Sestamibi scintigraphy and ⁶⁸Ga-Trivehexin PET/CT imaging. The agreement between ⁹⁹ᵐTc-Sestamibi scintigraphy and ⁶⁸Ga-Trivehexin PET/CT results was evaluated using the kappa coefficient for exploratory purposes within the scope of the proof-of- concept design of the study.

*The study protocol was approved by the Ethics Committee of Süleyman Demirel University, Faculty of Medicine Hospital (001, 13.09.2024). All procedures were carried out in accordance with the Helsinki Declaration.*

### ⁹⁹ᵐTc-Sestamibi parathyroid scintigraphy protocol

⁹⁹ᵐTc-Sestamibi parathyroid scintigraphy was performed with the SPECT Gamma Camera (Siemens Symbia Evo Excel; Siemens, Hoffman Estates, IL 60192, USA) according to the clinic’s routine practice protocol. Dual-phase planar images were acquired at 10 and 120 min following intravenous injection of 15–20 mCi (550–740 MBq) ⁹⁹ᵐTc-Sestamibi using a low-energy, high- resolution collimator. Mediastinal planar imaging and neck SPECT images were obtained at 60 min. The images were evaluated double-blind by two nuclear medicine specialists with at least 10 years of experience. Focal and persistent radiopharmaceutical uptake in late and/or SPECT images was considered a positive finding in favor of parathyroid adenoma.

### ^68^Ga-Trivehexin synthesis

⁶⁸Ga-Trivehexin synthesis was performed with a fully automated synthesis module (GRP model, Scintomics GmbH brand, Germany) in accordance with GMP standards. Disposable cationic purification cassettes and reaction kits provided by ABX GmbH (Germany) were used in the synthesis. The Trivehexin precursor (50 µg; Trimt GmbH, Germany) was labeled with ⁶⁸Ga eluted from a ⁶⁸Ge/⁶⁸Ga generator (iThemba Labs Ge/Ga68 Generator 1.85 GBq/50 mCi, South Africa). After labeling, C-18 cartridge-based purification was applied. The radiochemical purity of the final product was confirmed as over 95% by Radio-High Performance Liquid Chromatography (Scintomics GmbH, SCI HPLC System 8100Plus equipped with a Herm LB500 radionuclide detector, Germany).

### ⁶⁸Ga-Trivehexin PET/CT protocol

**Fig. 1:**
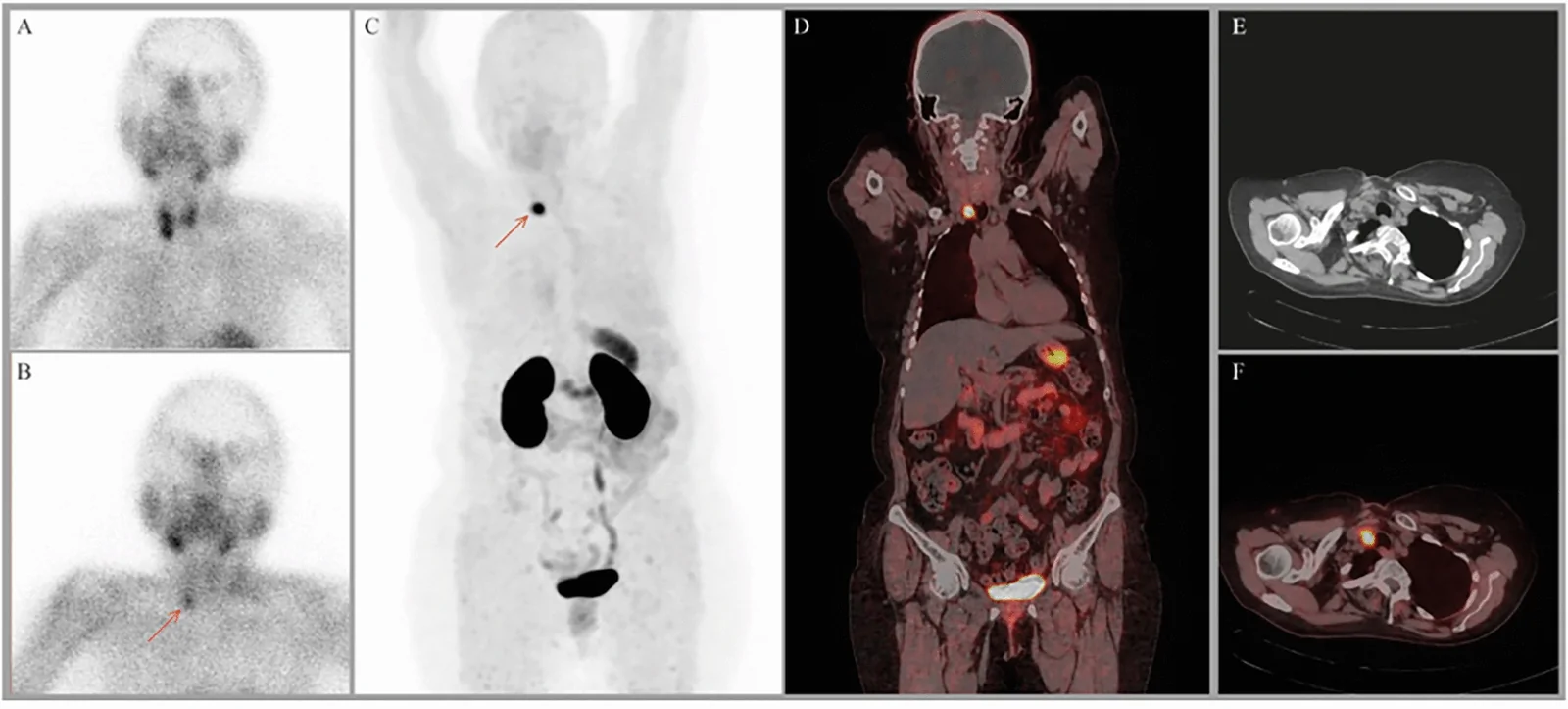
Concordant findings of parathyroid adenoma on ⁹⁹ᵐTc-sestamibi scintigraphy and ⁶⁸Ga-Trivehexin PET/CT. (**A**) Early-phase ⁹⁹ᵐTc-sestamibi SPECT gamma-camera image demonstrating a focal uptake in the right cervical region. (**B**) Delayed-phase ⁹⁹ᵐTc-sestamibi image showing persistent radiotracer retention at the corresponding right-sided site. (**C**) Maximum intensity projection (MIP) image of ⁶⁸Ga-Trivehexin PET/CT revealing intense focal uptake in the right neck region. (**D**) Fused PET/CT coronal image clearly localizing the radiotracer-avid lesion on the right side. (**E**) Corresponding axial CT image at the same anatomical level. (**F**) Fused PET/CT axial image demonstrating precise metabolic and anatomical correlation of the right-sided lesion

All patients underwent PET/CT imaging one week after a ⁹⁹ᵐTc-Sestamibi scan, followed by approximately 5 mCi (185 MBq) ⁶⁸Ga-Trivehexin injection one hour later. Images were acquired for 3 min in each bed position, from the crown of the head to the midpoint of the thigh. A General Electric Omni Legend PET/CT system (GE HealthCare, Haifa, Israel) was used for imaging. Focal increased radiopharmaceutical uptake in the area corresponding to the parathyroid lodge in the neck was considered positive. The maximum standardized uptake value (SUVmax), Trivehexin tumor volume (Trivehexin-TV), and total lesion Trivehexin (TL-Trivehexin) values were calculated in lesion-based analyses, and lesion diameters were measured from the CT images Fig. 1.

### Statistical analysis

The statistical analyses of the study were performed using SPSS 27.0 (IBM Inc., Armonk, NY, USA). Descriptive statistics were presented as Mean ± SD (Median; min–max) for numerical variables; frequency (percentage) for categorical variables. Cohen’s Kappa agreement coefficient was used for comparison of ⁹⁹ᵐTc-Sestamibi Scintigraphy and ⁶⁸Ga-Trivehexin PET/CT imaging methods in three separate diagnostic methods. In the analyses, a p-value < 0.05 was considered statistically significant at the 5% Type-I error rate.

## Results

The study was completed with a total of 28 patients evaluated for the diagnosis of parathyroid adenoma. A total of 64.2% of the patients were female, with a mean age of 56.14 ± 11.32 years (median: 55; min–max: 32–78). The mean serum Ca level was 11.33 ± 0.65 mg/dl, PTH level was 404 ± 571 pg/ml, and phosphorus (P) level was 2.18 ± 0.35 mg/dl (Table 1).

**Table 1:** Preoperative laboratory values and ⁶⁸Ga-Trivehexin PET/CT findings in patients with pathologically confirmed parathyroid adenoma after excisional biopsy

Measurement	Mean ± SD	Median	Min–Max
Parathyroid hormone (pg/ml)	404.10 ± 571.76	201	96–2154
Calcium (mg/dl)	11.33 ± 0.65	11	10.7–12.6
Phosporus (mg/dl)	2.18 ± 0.35	2.14	1.60–2.83
TL-Trivehexin (g) *	9.46 ± 6.04	8.7	2.1–22.8
Trivehexin-TV (cm^3^) **	2.50 ± 1.01	2.35	1.32–4.07
SUVMax	7.19 ± 5.71	5.28	2.02–23.62
Lesion diameter (mm)	10.81 ± 3.78	10	6–17

^*^Total Lesion Trivehexin

^**^Trivehexin Tumor Volume

Fine Needle Aspiration Biopsy (FNAB) was performed on 57.1% (n = 16) of the patients, and parathyroid cells were detected in 56.3% (n = 9) of these patients. PTH washout testing was performed on 60.7% (n = 18) of the patients, and high PTH levels were detected in 94.4% (n = 17) of these patients. A total of 13 patients (46.4%) underwent surgery. Histopathological evaluation revealed parathyroid adenoma in 12 patients and normal parathyroid tissue in 1 patient.

USI showed hypoechoic or suspected hypoechoic lesions consistent with parathyroid adenoma in all patients. ⁹⁹ᵐTc-Sestamibi scintigraphy yielded positive findings in 60.7% of patients (n = 17), while ⁶⁸Ga-Trivehexin PET/CT showed positive results. The positivity rate was found to be 78.6% (n = 22).

**Fig. 2:**
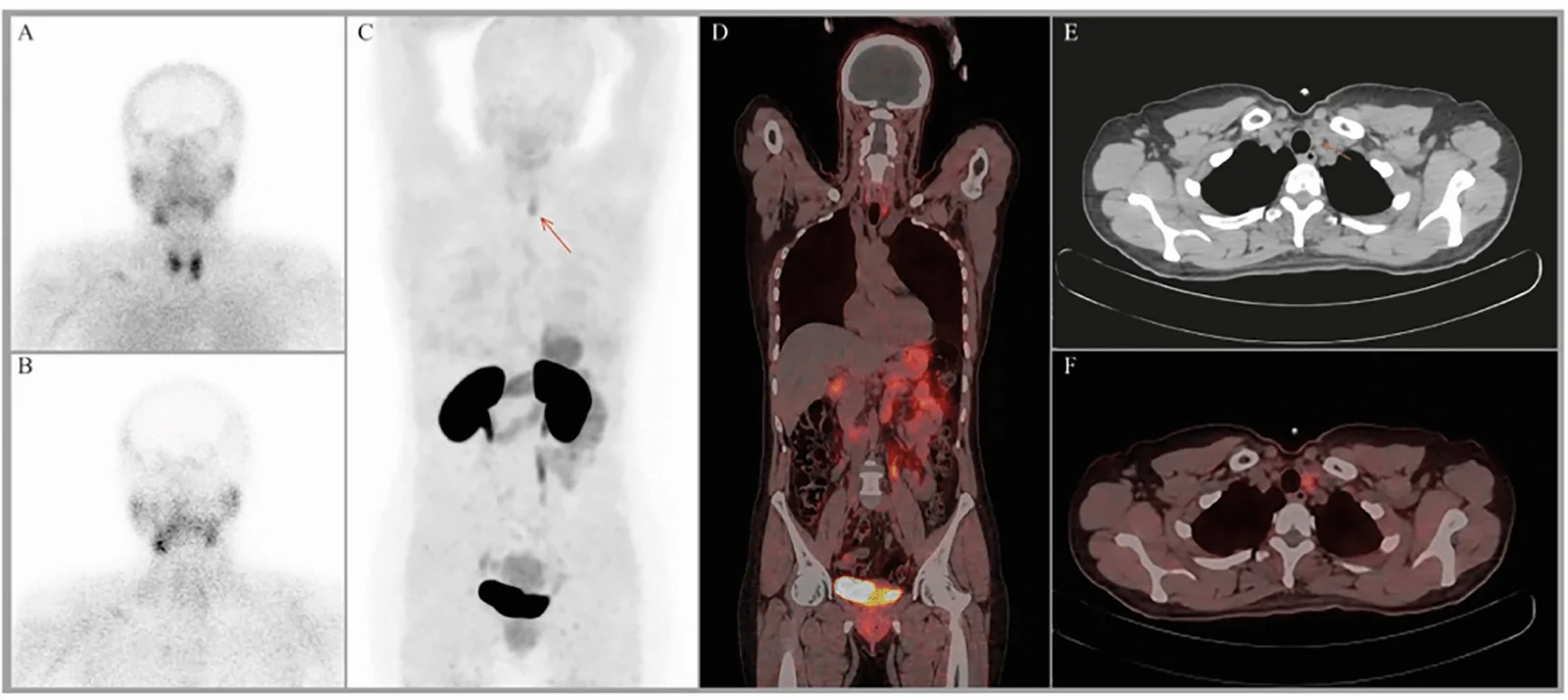
Parathyroid adenoma detected on ⁶⁸Ga-Trivehexin PET/CT in the absence of typical scintigraphic findings. (**A**) Early-phase ⁹⁹ᵐTc-sestamibi SPECT gamma-camera image demonstrating physiological thyroid uptake without abnormal focal uptake suggestive of parathyroid adenoma. (**B**) Delayed- phase ⁹⁹ᵐTc-sestamibi image showing no persistent focal uptake suggestive of parathyroid adenoma. (**C**) Maximum intensity projection (MIP) image of ⁶⁸Ga-Trivehexin PET/CT revealing a focal tracer uptake in the left cervical region. (**D**) Fused PET/CT coronal image showing focal radiotracer uptake corresponding to the left-sided lesion. (**E**) Corresponding axial CT image at the same anatomical level. (**F**) Fused PET/CT axial image confirming focal tracer uptake with precise anatomical localization

The mean lesion diameter measured on Ga-Trivehexin PET/CT was 10.81 ± 3.78 mm in 12 patients with histopathologically confirmed parathyroid adenoma. In these patients, the mean SUVmax value was calculated as 7.19 ± 5.71, Trivehexin-TV as 2.50 ± 1.01 cm3, and TL- Trivehexin as 9.46 ± 6.04 g (Table 1 and Fig. 2).

In patients who were diagnosed with parathyroid adenoma, 2 out of 3 patients (66.7%) with negative ⁹⁹ᵐSestamibi scintigraphy had a positive ⁶⁸Ga-Trivehexin PET/CT result. When all patients were evaluated, 6 out of 11 patients (54.5%) with negative ⁹⁹ᵐSestamibi scintigraphy had a positive ⁶⁸Ga-Trivehexin PET/CT result. In contrast, 16 out of 17 patients (94.1%) who had positive ⁹⁹ᵐSestamibi scintigraphy also showed positivity on ⁶⁸ Ga-Trivehexin PET/CT (Table 2).

**Table 2:** Comparison and kappa-based agreement between ⁶⁸Ga-Trivehexin PET/CT and ⁹⁹ᵐTc-sestamibi scintigraphy for the detection of parathyroid adenoma

		⁹⁹ᵐTc-sestamibi Scintigraphy N (%)		Kappa; p-value
		Negative	Positive	
⁶⁸Ga-Trivehexin PET/CT FNAB-positive (n = 9, %57.1)	Negative	1 (50.0)	0	0.609; p = 0.047*
	Positive	1 (50.0)	7 (100.0)	
⁶⁸Ga-Trivehexin PET/CT Washout-positive (n = 17, %94.4)	Negative	2 (28.6)	1 (10.0)	0.203; p = 0.323
	Positive	5 (71.4)	9 (90.0)	
⁶⁸Ga-Trivehexin PET/CT Excisional Biopsy- positive (n = 12, %92.3)	Negative	1 (33.3)	0	0.429; p = 0.070
	Positive	2 (66.7)	9 (100)	
⁶⁸Ga-Trivehexin PET/CT All Patients (n = 28, %100)	Negative	5 (45.5)	1 (5.9)	0.430; p = 0.013*
	Positive	6 (54.5)	16 (94.1)	

^*^: Statistically significant at the 0.05 level according to the kappa analysis FNAB: Fine needle aspiration biopsy

In patients with cells in the FNAB result, positive results were observed with ⁶⁸Ga-Trivehexin in 1 out of 2 patients (50%) who had a negative ⁹⁹ᵐTc-Sestamibi result. In those with elevated PTH in the washout, positive findings were observed with ⁶⁸Ga-Trivehexin in 5 out of 7 (71.4%) who had a negative ⁹⁹ᵐTc-Sestamibi result (Table 2).

In patients undergoing FNAB, statistically significant agreement was found between ⁹⁹ᵐTc- Sestamibi scintigraphy and ⁶⁸Ga-Trivehexin PET/CT results (Kappa: 0.609; p = 0.047). In patients undergoing washout, kappa values remained low between the two methods, and a statistically significant level of agreement was not reached (p > 0.05). The agreement between the two methods was moderate in patients with histopathologically confirmed parathyroid adenoma, but did not reach statistical significance (Kappa = 0.429; p = 0.070). According to the Landis & Koch scale, “moderate agreement” was observed. When all patients were evaluated, a statistically significant agreement was observed between Tc-99m Sestamibi scintigraphy and ⁶⁸ Ga-Trivehexin PET/CT (Kappa = 0.430; p = 0.013) (Table 2).

## Discussion

The present study evaluated the potential contribution of ⁶⁸Ga-Trivehexin PET/CT to parathyroid adenoma localization in patients suspected of primary hyperparathyroidism, in comparison with conventional ⁹⁹ᵐTc-Sestamibi scintigraphy. In light of the data, the study must be considered a proof-of-concept study to define the role of ⁶⁸Ga-Trivehexin PET/CT in parathyroid adenoma imaging. Experience with Trivehexin in parathyroid imaging is limited to a few studies in the literature, and our study presents one of the first clinical observations with this agent. Our results imply that ⁶⁸Ga-Trivehexin PET/CT can provide a higher lesion detection rate, particularly in Sestamibi-negative cases, and comes to the fore as a complementary imaging approach to conventional methods.

The detection of positive lesions with ⁶⁸Ga-Trivehexin PET/CT in a significant proportion of patients with negative ⁹⁹ᵐTc-Sestamibi scintigraphy may be consistent with the known limiting effects of mitochondrial content, oxidative metabolism, and cellular activity levels on Sestamibi uptake. In contrast, ⁶⁸Ga-Trivehexin’s ability to provide imaging via a different biological mechanism targeting αvβ6 integrin expression may provide an additional advantage in the detection of Sestamibi-negative parathyroid adenomas. The increasing importance of alternative imaging agents in Sestamibi-negative parathyroid adenomas is highlighted in the literature, and studies to evaluate the diagnostic contribution of different radiopharmaceuticals are still ongoing [9].

The wide variation in SUVmax, Trivehexin-TV, and TL-Trivehexin values obtained from Ga- Trivehexin PET/CT in patients with histopathologically confirmed parathyroid adenoma might be considered a result reflecting the biological and functional heterogeneity of parathyroid adenomas. This suggests that beyond evaluations based solely on visual positivity, semi-quantitative and volumetric parameters may have a potential role in parathyroid imaging.

The absence of reported diagnostic performance measures such as sensitivity and specificity in this study reflects a conscious methodological choice of an early-phase, exploratory (proof-of- concept) design. Because of the lack of histopathological confirmation across the entire patient group and the limited number of patients referred for surgery, it was anticipated that the calculated sensitivity values might not reflect the true diagnostic performance of the method. Also, the primary aim of our study was to demonstrate the clinical feasibility of Ga-Trivehexin PET/CT in imaging parathyroid adenomas. Rather than focusing on absolute diagnostic accuracy, the study aimed to evaluate intermethod agreement, and Kappa analysis was chosen as a more appropriate and reliable approach.

It was reported in the pioneering study published by Kuyumcu et al. that ⁶⁸Ga-Trivehexin PET/ CT provided a higher detection rate, especially in small parathyroid lesions that could not be detected by Sestamibi SPECT/CT, which was associated with the spatial resolution and lesion- background contrast advantages of PET-based imaging [10]. Similarly, in our study, it was found that ⁶⁸Ga-Trivehexin PET/CT could provide additional diagnostic contribution in cases evaluated as negative with conventional methods. However, because of the limited number of patients in both the existing literature and our study, and the fact that surgical-histopathological confirmation was not available for all cases, the findings obtained suggest that ⁶⁸Ga-Trivehexin PET/CT must be considered as an auxiliary imaging method in selected cases with difficult localization, rather than for routine clinical use.

In a recent review published by Ovcaricek et al., which discussed molecular imaging approaches in parathyroid carcinoma, it was reported that USI, ⁹⁹ᵐTc-Sestamibi scintigraphy, and 18F- fluorocholine PET/CT are fundamental modalities in clinical practice, but these techniques may show limitations, especially in biologically heterogeneous or atypical lesions. In the same review, attention was drawn to the potential role of PET agents targeting new molecular targets in cases where conventional imaging methods are insufficient, and it was stated that αvβ6 integrin-targeted 68Ga-Trivehexin PET/CT is a promising agent with limited clinical data [11]. Our study aimed to evaluate this approach, which is largely limited to case reports in the literature, and revealed the imaging performance of 68Ga-Trivehexin PET/CT in cases considered positive for parathyroid adenoma. Our findings suggest that integrin-targeted imaging could be a complementary alternative to mitochondria-based agents and could contribute to existing imaging algorithms, particularly in diagnostically challenging cases.

In the case report by Denizmen Zorba et al., it was argued that ⁶⁸^Ga-Trivehexin PET/CT detected recurrent lesions more sensitively than 1^⁸F-FDG PET/CT and ⁹⁹ᵐTc-Sestamibi SPECT/CT in recurrent parathyroid carcinoma. This highlights the potential advantage of αvβ6 integrin- targeted imaging in cases where FDG-negative or MIBI is limited [12]. In our study, the successful localization of ⁶⁸Ga-Trivehexin PET/CT in non-malignant parathyroid adenoma cases suggests that this agent may be a complementary imaging method not only in recurrent or aggressive parathyroid diseases but also in primary hyperparathyroidism.

In another recent case report published by Kar et al., it was reported that in a patient with primary hyperparathyroidism, ⁶⁸Ga-Trivehexin PET/CT showed significant uptake in parathyroid adenoma and lower uptake in brown tumors compared to FDG [13]. This suggests that Trivehexin might reflect increased αvβ6-integrin expression in parathyroid adenomas, which is consistent with the high localization success obtained in our study.

Kumar et al. drew attention to the potential of ⁶⁸Ga-Trivehexin PET/CT to visualize parathyroid tissue by demonstrating parathyroid hyperplasia and accompanying widespread skeletal involvement in a case of secondary hyperparathyroidism [14]. In our study, it was found that ⁶⁸Ga- Trivehexin PET/CT could show targeted uptake in patients suspected of primary hyperparathyroidism, where conventional methods were limited, and these findings provide preliminary data on the clinical use of the agent.

In addition to these findings, Benjamin et al. reported that ^1^⁸F-fluorocholine PET/CT demonstrates high diagnostic performance in the localization of parathyroid adenomas, particularly in cases where conventional imaging methods are inconclusive [15]. In the present study, we highlighted the potential of ⁶⁸Ga-Trivehexin, a novel PET radiotracer, for the visualization of parathyroid tissue. In this context, further prospective and comparative studies directly evaluating the diagnostic performance of these two PET radiotracers are warranted.

In this context, the results suggest that ⁶⁸Ga-Trivehexin PET/CT might offer an alternative approach based on biological targeting in parathyroid imaging, and further studies are needed to identify which patient groups would benefit most from it, especially in clinical scenarios where conventional methods produce limited data.

## Limitations

The study had some limitations. Firstly, the single-center design and limited number of patients restricted the generalizability of our results. However, this is consistent with the proof-of-concept nature of the study. The ⁹⁹ᵐTc-Sestamibi imaging used in our study was performed with only a planar/SPECT gamma camera instead of a SPECT/CT hybrid system, and the lack of anatomical correlation provided by the CT component might have limited the diagnostic performance of scintigraphy, especially in the localization of small or atypical lesions. Also, histopathological confirmation was not available in all patients. In cases where surgery was not performed, Fine Needle Aspiration Biopsy (FNAB) cytology and/or PTH washout tests were used for diagnostic confirmation, which might have caused a possible confirmation bias. Diagnostic performance measures such as sensitivity and specificity were not calculated because of the limited sample size, and comparisons between imaging methods were evaluated only with agreement analysis (kappa coefficient).

## Conclusion

This proof-of-concept study implies that ⁶⁸Ga-Trivehexin PET/CT is a feasible and promising imaging method for localizing parathyroid adenomas in patients with primary hyperparathyroidism. In particular, the positive findings of ⁶⁸Ga-Trivehexin PET/CT in some cases where lesions could not be detected with ⁹⁹ᵐTc-Sestamibi scintigraphy suggest that this method could be a complementary diagnostic tool. However, further multicenter, advanced phase studies with larger patient populations are needed to confirm these findings and to determine the clinical use of ⁶⁸Ga- Trivehexin PET/CT.
